# The Nervous System and Its Functions

**Published:** 1842-10

**Authors:** 


Art. I.—*The Nervous System and its Functions.* By Herbert Mayo, F.R.S., Senior-surgeon of the Middlesex Hospital, &c.—*London*, 1842. 12mo, pp. 182.

This little treatise was originally intended as a companion to a new edition of the author's Engravings of the Brain; but after commencing it, he thought it preferable to publish it separately in an expanded form. It is characterized by the clearness and precision which mark all the author's statements; and which, when brought to bear upon abstract truths, renders his exposition of them so lucid and valuable. This is especially the case in the introductory remarks on the relation of vitality to mind; the whole of which we should be glad to quote, as expressing a great deal of profound thought in a form in which elegance and conciseness are remarkably united. The following we consider as a fair specimen of the whole:

"Life is a force so contrived and used as to qualify the materials of the inert world for a temporary union with consciousness; a means how mind may enter into such relations with matter, that it may have its being and part in physical nature, and its faculties developed, and its capabilities and tendencies drawn out and proved (for whatever ulterior purpose) in subjection to, and in harmony with her laws. As we imagine the Supreme Mind to be ubiquitous, infinite, controlling but uncontrolled by matter, so, in contrast with these attributes, we conceive the finite mind to be bound down to place, and to be dependent on a certain arrangement of matter for its manifestation—each power displayed as the property of a tissue, each agency as the function of an organ. These views do not lead to materialism. For one cannot disjoin the physiology of the nervous system from mental philosophy, nor investigate the play of its organs without attending to the mind itself. And if equal consideration is given to the two classes of phenomena, it is impossible (so, at least, it appears to myself) to avoid the conviction that they are essentially independent the one of the other, and belong to distinct essences; and that *ipseity*—the consciousness of personal being—is not a mode of material existence, nor physical impenetrability an attribute of that which feels and thinks." (pp. 6-7.)

We are sorry that we cannot view the rest of the work as favorably. We do not find fault with its form and style, for we consider this peculiarly well adapted to its purpose. The author has arranged his materials in the form of isolated propositions, to each of which is appended a demonstration or illustration; and, when taken in connexion with each other, these propositions alone give a concise view of the doctrines he professes. What we regret to have so continually forced on our notice is that peculiar form of ipseity, termed egotism, which seems to be a besetting sin too common amongst scientific men, leading them to set a disproportionate value on their own achievements, and to under-estimate those of every one else. Yet we should not impute it to *all;* for the

*most* distinguished men of every age have been those most willing to award to others the merit which was justly due to them.

Any one who is acquainted with the history of discovery in the nervous system during the last twenty-two years, must be well aware that no one is likely to arrive at an accurate estimate of what has been actually accomplished, who does not go into the investigation with an entire absence of all party spirit, and with a thorough desire to seek truth wherever it may be found. That *we* have endeavoured to pursue this course, we think our readers have had ample evidence. How far Mr. Mayo is free from prejudice, may be judged from the following sentence, which, as far as we can perceive, is the only reference to Dr. Marshall Hall throughout the whole of this treatise on the nervous system: "Dr. M. Hall has given the good name of reflex function to this circle of impression and action, and has added one or two interesting facts in additional illustration of the principle." (p. 50.) We too clearly perceive, also, the same desire to deprive Sir C. Bell of as much merit as possible, not only in his own favour, but in that of Magendie, on which we commented with regret on a former occasion. Mr. Mayo still maintains, in spite of what we believe has been generally felt to be the clear exposition of the question which we gave in our Ninth Volume (p. 131), that Sir C. Bell, in his paper of 1821, "had as yet made no advance towards the conception of different nerves being appropriated to sense and motion;" and that the establishment of the true doctrine of the functions of the nerves of the face is due to himself, and that of the nerves of the spine to Magendie. Thus we find him stating that Magendie, in his paper of 1822 in the Journal de Physiologie, "incontrovertibly established that the anterior roots of the spinal nerves minister to voluntary motion *exclusively*, the posterior to sensation;" a doctrine of which almost the very reverse was maintained by Magendie for many years afterwards, (See Vol. IX. p. 134.) It is due to Mr. Mayo, however, that, as we formerly inserted Mr. A. Shaw's statement of the relation which once subsisted between Sir C. Bell and himself, we should now give place to his own. Which is the more correct, we shall leave those to decide who are specially interested in this piece of personality. After speaking of Sir C. Bell's essay privately distributed in 1811, he continues:

"It was a step of eminent service to science then, and again later in his career of investigation, to set on foot new inquiries into the functions of the nerves, and to show that they might be elucidated by new experiments. With the researches of Sir Charles Bell, made in this his first essay, and up to this point, I had the advantage of being familiar, having been his pupil in the years 1812, 1813, 1814, and 1815. After this I was not in the way of knowing, nor did I know anything whatever of the nature of Sir Charles Bell's researches, except through his published writings." (p. 37.)

How far this statement is consistent with the acknowledgment of assistance derived from Mr. Mayo, in three of Mr. J. Shaw's papers published in 1821 and 1822, we cannot clearly perceive.

We shall not dwell upon the scientific portion of Mr. Mayo's treatise, for the simple reason that to do so would require a great deal of unprofitable discussion, the minds of physiologists being too much made up in regard to certain leading doctrines, to be disturbed by the dogmatic and often contradictory assertions of Mr. Mayo. We may simply say that the same inconsistencies which we formerly pointed out in his doctrines (Vol. V. p. 534,) still remain, and are even rendered more palpable. Thus we find him allowing in one place that the movements in the limbs of a decapitated frog do not depend upon sensation, (pp. 20-1;) whilst he afterwards adduces actions of a similar kind to prove that sensibility and even voluntary power exist in the spinal cord, provided its connexion with the segment of the medulla oblongata, from which the fifth pair arises, be entire. The actions of acephalous infants he considers as being thus directed: and again, in a subsequent page (54) he enunciates the proposition that "in the entire and living frame, each segment of the cranio-spinal cord, with its nerves, constitutes the whole apparatus requisite and sufficient for sensation being felt, and the impulses to muscular motion, both reflex and conscious, originated." To reconcile these and many other contradictions so completely surpasses *our* ability, that we must leave the question in the hands of our readers.

